# Validation of the Bahasa Malaysia Version of the General Attitudes Towards Artificial Intelligence Scale Among Academicians

**DOI:** 10.21315/mjms-05-2025-336

**Published:** 2025-10-31

**Authors:** Nur Ain Azman, Nur Syahmina Rasudin, Rohani Ismail, Noraini Abdul Ghafar, Shamarina Shohaimi, Nor Shafrin Ahmad, Norsiah Abdul Hamid

**Affiliations:** 1 School of Health Sciences, Health Campus, Universiti Sains Malaysia, Kelantan, Malaysia; 2 Department of Biology, Faculty of Science, Universiti Putra Malaysia, Selangor, Malaysia; 3 School of Education Studies, Universiti Sains Malaysia, Pulau Pinang, Malaysia; 4 School of Multimedia Technology and Communication, Universiti Utara Malaysia, Kedah, Malaysia

**Keywords:** artificial intelligence, academicians, attitudes, psychometrics, questionnaire, validation study

## Abstract

**Background:**

The growing incorporation of artificial intelligence (AI) within academic settings has prompted increased scholarly attention to understanding how academicians perceive its impact. Although the General Attitudes Towards Artificial Intelligence Scale (GAAIS) is a widely recognised instrument for evaluating perceptions of AI, a formally validated version in Bahasa Malaysia has not yet been developed. This study, therefore, sought to adapt and validate the GAAIS in Bahasa Malaysia to assess attitudes toward AI among Malaysian academicians accurately.

**Methods:**

A cross-sectional study was conducted among academicians at the Universiti Sains Malaysia Health Campus in Malaysia. A Confirmatory Factor Analysis (CFA) was performed to evaluate the factor structure of the translated scale. Composite reliability (CR) was utilised to assess reliability, whereas convergent validity was determined by calculating the average variance extracted (AVE).

**Results:**

The final CFA model confirmed a two-factor structure, retaining 14 out of 20 original items. Factor 1 (Optimism and Benefits) comprised eight items, while Factor 2 (Risks and Ethical Concerns) consisted of six items. The model demonstrated strong fit indices (*χ*^2^/df = 1.616, TLI = 0.947, CFI = 0.936, RMSEA = 0.073) and high internal consistency (CR values: Factor 1 = 0.899, Factor 2 = 0.888). An AVE of 0.530 for Factor 1 and 0.573 for Factor 2 indicates the convergent validity of the scales.

**Conclusion:**

The translated Bahasa Malaysia version of GAAIS is a valid and reliable tool for assessing AI attitudes in general among Malaysian academicians. Its relevance spans multiple academic fields and professional industries, contributing valuable support to ongoing research involving multilingual and multicultural populations.

## Introduction

Artificial Intelligence (AI) has rapidly advanced technologically, impacting many areas of society such as education, science, the economy, and politics (1–4). It has become an integral part of most technologies, devices, and systems, improving efficiency, decision-making, and automation across various industries (5). AI aims to streamline operations and enhance user experiences, as evidenced by its use in automated driving, medical diagnosis, human resource management, judicial processes, food industries, and agriculture (6–11). People have mixed perceptions about integrating AI into their daily lives. Many view AI as revolutionary because it quickly exceeds human capabilities by solving complex problems, reducing errors, and opening up new opportunities (12, 13). These advancements represent AI’s anticipated benefits, which are expected to foster positive attitudes toward its adoption.

Despite the growing optimism towards AI, people also express concerns about its potential risks and moral implications (14). Automation in industries raises the risk of job displacement, especially for routine tasks, as it aims to improve efficiency and cut labour costs (15, 16). AI systems are routinely tasked with processing extensive datasets, and as reliance on such technologies continues to expand, so too does public and institutional concern regarding issues of data privacy and information security (17). Unauthorised access and data breaches heighten concerns about the misuse of personal and sensitive information, damaging public trust in AI systems (18). Individuals might also resist accepting AI innovations, particularly those who are already accustomed to traditional methods (19). People might also feel pressured to adopt these technologies because workplace policies encourage it, as companies aim to stay competitive in their industries (20).

AI is also actively integrated into the Malaysian education sector, where academicians in public universities adopt AI in research, teaching and administrative management (21, 22). The efficiency and academic productivity of academics are enhanced using AI-powered tools to assist in curriculum planning, research data analysis, automated grading and plagiarism detection (23–25). While a number of academics recognise the potential of AI as a resource for teaching and research, others remain sceptical, frequently due to reluctance toward innovation or limited experience with AI technologies (26). Apart from its practical uses, employing AI in academia raises important ethical issues, especially regarding academic integrity (27, 28). The integration of AI into academic work presents complex challenges to the authenticity of human intellectual contributions, particularly to accountability, authorship, and transparency (2, 29). AI poses a tangible risk of enabling plagiarism and the inappropriate handling of sensitive academic data. However, as these systems cannot be held accountable for the content they generate, the onus remains on academic institutions and individual users to ensure ethical and responsible usage (21, 30). There are reported issues about academic papers written completely using ChatGPT, sparking debates on how content generated by AI tools affects the credibility standards in academia (31, 32).

Varied attitudes toward AI adoption underscore the importance of developing a robust assessment tool to gauge individual perceptions of AI at the personal level accurately. Several assessment tools have been designed to measure attitudes related to AI, such as the Artificial Intelligence Literacy Scale, which assesses user competence; the Artificial Intelligence Anxiety Scale, which examines fear and emotional responses; and the Attitude Towards Artificial Intelligence – Short Measure (ATAI), which gauges general sentiment toward AI (33–35). However, these instruments are limited to specific constructs; i.e., they operate within narrowly defined conceptual parameters and may not be effective or applicable beyond those limits. In contrast, the General Attitude Towards Artificial Intelligence Scale (GAAIS) provides a more balanced and comprehensive framework by capturing both positive and negative perceptions of AI. This dual factor structure, developed by Schepman and Rodway, makes the GAAIS suitable for measuring general attitudes toward AI among individuals (36). It is a standardised 20-item scale that measures individuals’ perceptions, concerns, and acceptance of AI.

The scale has been applied across various populations and translated into several languages, including Korean, German, and Turkish (37–39). The questionnaire has yet to be translated into Bahasa Malaysia, the official language of Malaysia, nor has it been subjected to validation procedures to ensure its linguistic and cultural suitability. A validated Bahasa Malaysia version of GAAIS is necessary to ensure that AI attitude assessment remains accurate and meaningful. This study was conducted to validate the Bahasa Malaysia version of GAAIS for assessing academicians’ attitudes towards AI in Malaysia, using Confirmatory Factor Analysis (CFA).

## Methods

### Research Design

This research adopted a quantitative design, employing a cross-sectional survey to validate a 20-item questionnaire assessing academicians’ attitudes toward AI in public universities in Malaysia. A cross-sectional design was selected because it is often used in early validation research to examine factor structure and internal consistency at one time point (40). According to Walter et al. (41) and Mundfrom et al. (42), a sample size exceeding 100 was considered adequate for factor analysis and reliability assessment to produce statistically meaningful results. The most frequently used method for determining sample sizes in validation studies was based on the item ratio (43). The sample size for this study was determined using a 6:1 variable ratio, with an allowance for a 20% potential drop-out, resulting in a requirement of 144 participants. Ultimately, 116 valid responses were collected, corresponding to a response rate of 80.5%.

The structured questionnaire was administered to academic lecturers from Health Campus, Universiti Sains Malaysia, Kelantan, via Google Forms through their email addresses from September 2024 to November 2024. Participants were selected using simple random sampling from the name list provided by the administration office to ensure unbiased representation of the target group. The inclusion criteria specified local academicians who served as either permanent or contract lecturers and possessed at least a tertiary-level qualification. Academicians in top management positions were excluded, as their job responsibilities and workloads differ from those of teaching lecturers. Follow-up reminders were sent periodically over 10 days to improve the response rate.

All procedures involving research study participants were approved by the Human Research Ethics Committee of Universiti Sains Malaysia with approval code USM/JEPeM/KK/24010097. All methods were performed in accordance with relevant guidelines and regulations, including the Declaration of Helsinki. Participation in this study was voluntary, and all participants were fully informed of their right to withdraw from the study, as well as the anonymity and confidentiality of their responses to protect their privacy. Informed consent was obtained from all participants before their involvement in the study.

### Research Instrument

The research applied the GAAIS, developed by Schepman and Rodway to measure individuals’ general attitudes towards AI (36). This instrument consisted of 20 items classified into two domains: Positive GAAIS (12 items) to measure favourable attitudes toward AI, including benefits and trust in AI applications, and Negative GAAIS (8 items) to measure concerns related to AI risks, ethical implications, and threats. Translation and back-translation of the instrument into Bahasa Malaysia were done by translators from the School of Languages, Literacies and Translation at Universiti Sains Malaysia, in accordance with international guidelines. The translated questionnaire was pre-tested among a small group of academicians to improve its readability, formatting, and time taken to complete the questionnaire.

As the instrument was employed in its original form without any modifications to its items, an assessment of content validity was not undertaken in this study. The response options for each item were assessed using a five-point Likert-type rating scale (5 = strongly agree, 4 = agree, 3 = neither agree nor disagree, 2 = disagree, 1 = strongly disagree). Negative GAAIS items were reverse-coded to maintain consistency in score interpretation.

### Statistical Analysis

The descriptive statistics were analysed using IBM SPSS Statistics software (version 28.0). The mean and standard deviation were determined for continuous variables, while frequency and percentage were used to summarise categorical variables.

Construct validity was evaluated through CFA using IBM SPSS AMOS (version 24.0) to test a predefined theoretical model of how observed variables connect to underlying latent factors. Because the questionnaire was developed with specific subfactors and no items were altered, construct validity assessment did not involve Exploratory Factor Analysis.

The model fitness was evaluated using the following indices to indicate a good fit: Chi-square goodness-of-fit (*χ*^2^/df) < 3.0, the Comparative Fit Indexes (CFI) > 0.90, the Tucker-Lewis Indexes (TLI) > 0.90, and root mean square error of approximation (RMSEA) < 0.08 (44). The average variance extracted (AVE) was set at > 0.50, and factor loadings were set at > 0.50. Composite reliability (CR) was used to confirm the reliability of each construct with a minimum value of 0.7, which was considered a satisfactory level for the parameter (44). Internal consistency was further evaluated using Cronbach’s alpha, with a threshold of ≥ 0.70 deemed acceptable for reliability (44). Discriminant validity was assessed using the Fornell–Larcker criterion, which compares each construct’s square root of the AVE against its correlations with other constructs. The results demonstrated that each construct’s AVE square root exceeded its inter-construct correlations, confirming an acceptable level of discriminant validity within the measurement model (45).

## Results

### Sociodemographic Characteristics

A total of 116 participants completed the questionnaire. The majority of participants were female (67.2%) and Malay (94.8%). The mean age of the participants was 44.37 years (SD = 6.77), with 89.7% being married. Nearly all participants were from the School of Medical Sciences (54.3%) and the School of Health Sciences (34.5%). Regarding academic positions, 44.0% of individuals were lecturers and 37.1% were senior lecturers. The sample included 50.9% individuals with a master’s degree and 49.1% with a PhD. Table 1 presents a comprehensive demographic breakdown.

### CFA

CFA of the initial model showed that 14 items had factor loadings more than 0.50. Four items (AI9, AI11, AI12 and AI13) from Factor 1 (Positive GAAIS) and two items (AI10 and AI14) from Factor 2 (Negative GAAIS) had low factor loadings (< 0.50) and were removed.

**Figure 1:**
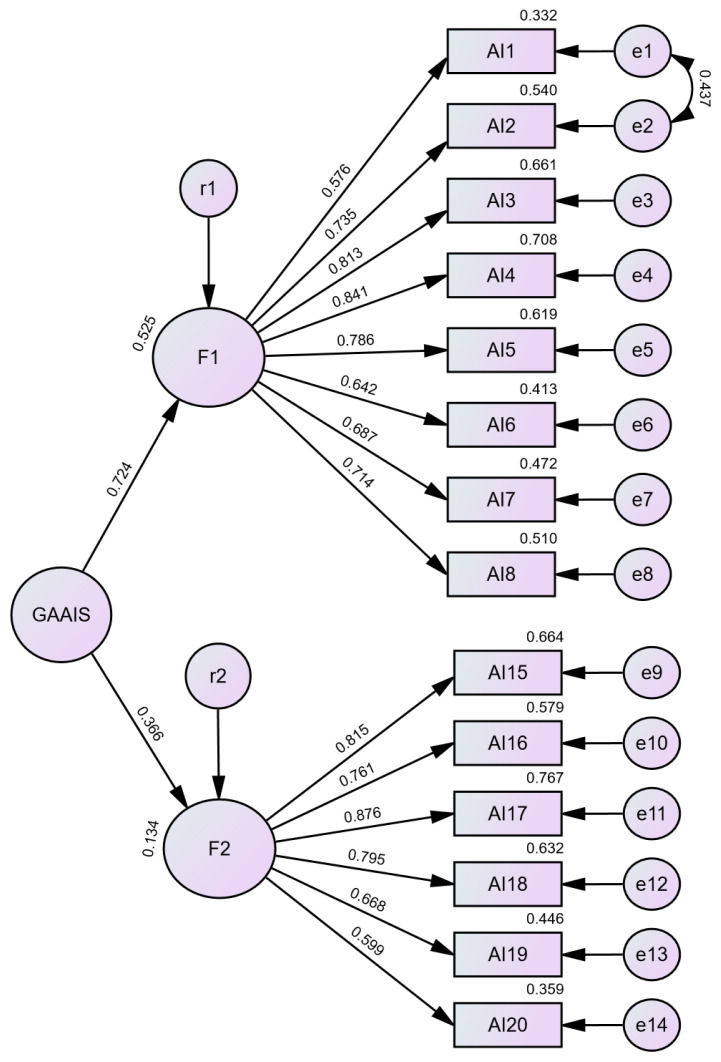
Final model of Confirmatory Factor Analysis The model demonstrates a two-factor structure with 14 retained items: Factor 1 (Optimism and Benefits, eight items) and Factor 2 (Risks and Ethical Concerns, six items). All standardised factor loadings exceed the 0.50 threshold, supporting construct validity. The model fit indices indicate good fit (*χ*^2^/df = 1.616, CFI = 0.936, TLI = 0.947, RMSEA = 0.073).

After removing the misfitting items, the revised CFA model was tested for goodness-of-fit. Although the factor loadings exceeded 0.50, the model demonstrated a poor fit overall. The *χ*^2^/df value was within an acceptable range at 1.869, along with satisfactory CFI (0.924) and TLI (0.909) values. However, the RMSEA was 0.087, surpassing the recommended threshold of 0.08. To improve the model fit, redundant items AI1 and AI2 were identified as having high value modification indices (MI = 19.332). The final revised model (Figure 1) exhibited an enhanced fit, with each item retaining a loading factor exceeding the 0.5 threshold. The model adequately fits the data with indices *χ*^2^/df = 1.616, CFI = 0.947, TLI = 0.936 and RMSEA = 0.073.

The CR values for the two factors were 0.899 and 0.888, exceeding the recommended threshold of 0.70, indicating acceptable internal reliability among the items. Similarly, Cronbach’s alpha values of 0.898 for Factor 1 and 0.886 for Factor 2 further supported strong internal consistency. The AVE values were 0.530 and 0.573, surpassing the standard cut-off of 0.50, thus demonstrating adequate convergent validity. Discriminant validity was established via the Fornell–Larcker criterion, where the square roots of AVE for Factor 1 (0.728) and Factor 2 (0.757) were both greater than the inter-factor correlation (0.265), indicating that each construct was empirically distinct. In summary, the translated GAAIS model demonstrated satisfactory reliability and validity metrics for evaluating attitudes toward AI (see Table 2).

## Discussion

This study aimed to translate and validate the GAAIS into Bahasa Malaysia to assess Malaysian academicians’ attitudes toward AI (36). The final, revised version of the GAAIS, translated into Bahasa Malaysia, was validated through the study’s findings. This version retained 14 of the original 20 items, and the validated structure revealed two distinct factors: one representing positive attitudes toward AI and the other representing negative attitudes toward AI.

The study’s sample size was 116 academicians from a healthcare background, dominated by female respondents. This reflected a common trend seen in healthcare-related studies: Women constitute a significantly large proportion of the healthcare workforce, especially in fields like medicine, nursing, and allied health (46). The academicians’ ages ranged from 31 to 67, with an average of 44.37. Most participants held lecturer or senior lecturer positions. These findings may indicate that a substantial proportion of faculty members are mid-career or that younger individuals were more likely to participate in this research study than older individuals. Most participants were Malay, reflecting the demographic distribution of government-funded institutions, especially in Kelantan, where the Malay population is the largest.

While female and Malay academicians dominated the sample from a single health sciences campus, this does not limit the scale’s generalisability. The instrument was designed to assess general attitudes toward AI rather than attitudes based on demographic traits such as gender or ethnicity. Its factor structure and item content were designed to be neutral and applicable to a broad range of groups (36).

CFA was conducted to validate the factor structure of the translated items. During the initial analysis, six items failed to load above 0.50 and were therefore removed from the analysis. Specifically, Item AI9 (For routine transactions, I would rather interact with an artificially intelligent system than with a human), along with Items AI11 (An artificially intelligent tool would be better than an employee in many routine jobs), AI12 (I would like to use Artificial Intelligence in my own job), and AI13 (Artificially intelligent systems can help people feel happier), all categorised under Factor 1 (positive attitude towards AI) demonstrated factor loadings of 0.137, 0.199, 0.401, and 0.456, respectively. Additionally, Items AI10 (Organisations use Artificial Intelligence unethically) and AI14 (I think artificially intelligent systems make many errors), which are associated with Factor 2 (negative attitude towards AI), had factor loadings of 0.325 and 0.398, respectively. These values did not meet the acceptable threshold, so the items were excluded from further analysis.

The model was reanalysed with items that had factor loadings above 0.5, and the model fit remained good (*χ*^2^/df ratio = 1.862, CFI = 0.924, TLI = 0.909), except for RMSEA (0.087), which slightly exceeded the recommended range of 0.08. To improve the model fit, redundant items were identified using high value MI with the suggested value below 15 (47). Only one pair of items, AI1 (There are many beneficial applications of Artificial Intelligence) and AI2 (I am impressed by what Artificial Intelligence can do), showed a high MI value of 19.332. Both items express a general positive appraisal of AI’s capabilities, which may lead to unexplained variance. Thus, correlating their error terms is justified, as both items likely evoke similar cognitive responses related to optimism toward AI.

The model was revalidated until the final analysis indicated that the model fit the data appropriately. The final model showed a good fit, with *χ* ^2^/df = 1.616, TLI = 0.947, CFI = 0.936, RMSEA = 0.073, and the highest MI decreased to 9.705. The model’s reliability was assessed for both factors using CR, which evaluates the consistency of items within each factor. The CR values were 0.899 for Factor 1 and 0.888 for Factor 2, both exceeding the commonly recommended threshold of 0.7. Cronbach’s alpha values for both factors (Factor 1 = 0.898, Factor 2 = 0.886) also exceeded 0.7, confirming strong internal consistency. Furthermore, the AVE from both factors surpassed the suggested 0.50 threshold, indicating adequate convergent validity of the model construct. Notably, discriminant validity was demonstrated through application of the Fornell–Larcker criterion, as the square roots of the AVE exceeded the interfactor correlations. This result affirms that the two constructs are statistically distinct.

These results confirmed that the final translated GAAIS model was both valid and reliable as a tool for assessing AI attitudes among Malaysians. The original authors classified the model structure into positive and negative attitudes toward AI. The factor labels should be updated to better align with the conceptual and theoretical framework of the model, considering the items that were removed. Factor 1, which included AI1 through AI8, indicating positive perceptions and benefits of AI, will now be named “Optimism and Benefits.” Meanwhile, Factor 2, comprising AI15 through AI20, reflecting fears, scepticism, and ethical issues, will be renamed “Risk and Ethical Concerns.”

As previously noted, six items were removed due to low factor loadings. Compared to previous validations conducted in Korean and Turkish populations, this study required a greater degree of item reduction (37, 38). The underperformance items in this study may be attributed to limited contextual relevance and semantic ambiguity (48). For instance, items AI9 and AI11, which imply a preference for automation over human interaction, may have posed interpretive challenges for respondents. This difficulty likely stems from many respondents lacking direct experience with AI technologies in their service environments. As a result, their ability to meaningfully assess or respond to these items may be compromised. Item AI12, which presumes the use of AI in professional tasks, may not have resonated with participants, as many do not frequently employ AI in their academic activities. AI10 and AI14 may have demonstrated suboptimal performance due to their broadly worded phrasing. The generalised nature of these items may have contributed to participants’ unfamiliarity with contentious or nuanced applications of AI, thereby leading to inconsistent interpretations and reduced response reliability. Furthermore, item AI13 exhibits high emotional abstraction, which may lead to limited semantic clarity and potential irrelevance within an academic setting.

Although six items were excluded, their removal does not appear to compromise the instrument’s generalisability, as the remaining items continue to represent the core construct of attitudes towards AI. The scale maintains its conceptual balance by covering both optimistic and critical perspectives. The excluded items were removed due to insufficient contextual relevance, clarity, or direct applicability to participants’ experiences. Nevertheless, the remaining items aligned distinctly with the initial two-factor structure and preserved comprehensive coverage of general attitudes toward AI. Accordingly, the theoretical integrity of the GAAIS is maintained, as evidenced by the robust reliability and validity metrics associated with the retained structure.

This study significantly contributed to bridging the language gap in AI research by introducing the validated Bahasa Malaysia version of the GAAIS assessment tool. It enhanced accessibility for non-English speakers to engage in AI-related discussions, promoting broader participation and facilitating a more diverse understanding of AI across various disciplines. This research focused on academic professionals and highlighted the importance of AI within educational environments, underscoring its impact on AI awareness that extends beyond traditional technical fields such as information technology and engineering. The questions in the GAAIS aim to measure general attitudes towards AI, ensuring their relevance across diverse fields. This study had certain limitations. For example, it did not measure participants’ familiarity with AI, which may have affected their attitudes toward adopting AI. Assessing individuals’ prior exposure to AI may yield valuable insights into the formation of perceptions and facilitate the development of more effective, targeted AI-related interventions. Furthermore, criterion-related validity, such as the ability of GAAIS scores to predict actual AI usage or behaviour, was not evaluated and warrants future consideration.

## Conclusion

The GAAIS was effectively translated into Bahasa Malaysia and subjected to validation procedures, demonstrating its reliability and validity for evaluating attitudes toward AI among Malaysian academicians. The final two-factor CFA model retained 14 out of 20 items, with factor loadings greater than 0.50, and demonstrated strong model fit, internal consistency and reliability. Therefore, the translated Bahasa Malaysia version of GAAIS is a robust instrument for understanding perceptions of AI’s opportunities and risks among academicians and across various fields. Further studies may be conducted to assess the validity of the scale measuring attitudes towards AI across different academic disciplines and industry sectors, thereby improving the generalisability of findings in higher education and professional contexts.

## Figures and Tables

**Table 1:** Sociodemographic characteristics of participants (*N* = 116)

Variable	Frequency (%)	Mean (SD)
**Gender**		
Male	38 (32.8)	
Female	78 (67.2)	
**Age (years)**		44.37 (6.769)
< 40	30 (25.9)	
40 to 60	85 (73.2)	
> 60	1 (0.9)	
**Ethnicity**		
Malay	110 (94.8)	
Chinese	6 (5.2)	
**Academic position**		
Lecturer	51 (44)	
Senior lecturer	43 (37.1)	
Associate professor	18 (15.5)	
Professor	4 (3.4)	
**Faculty**		
School of Medical	63 (54.3)	
Sciences		
School of Health Sciences	40 (34.5)	
School of Dental Sciences	13 (11.2)	
**Education level**		
Master’s degree	59 (50.9)	
PhD	57 (49.1)	
**Marital status**		
Single	9 (7.8)	
Married	104 (89.7)	
Divorced/widowed	3 (2.6)	

SD = standard deviation

**Table 2:** The result of factor loadings and reliability of measurement model

Construct	Item	Item description	Outer loadings (standardised)		CR

			Initial model	Modified model	
Positive GAAIS (F1)	AI1	There are many beneficial applications of Artificial Intelligence	0.614	0.576	0.899
	AI2	I am impressed by what Artificial Intelligence can do	0.746	0.735	
	AI3	Artificial Intelligence can have positive impacts on people’s wellbeing	0.806	0.813	
	AI4	Artificial Intelligence is exciting	0.827	0.841	
	AI5	Artificial Intelligence can provide new economic opportunities for this country	0.777	0.786	
	AI6	Artificially intelligent systems can perform better than humans	0.638	0.642	
	AI7	Much of society will benefit from a future full of Artificial Intelligence	0.696	0.687	
	AI8	I am interested in using artificially intelligent systems in my daily life	0.726	0.714	
	AI9	For routine transactions, I would rather interact with an artificially intelligent system than with a human	0.137	Removed	
	AI11	An artificially intelligent tools would be better than an employee in many routine jobs	0.199	Removed	
	AI12	I would like to use Artificial Intelligence in my own job	0.401	Removed	
	AI13	Artificially intelligent systems can help people feel happier	0.456	Removed	

Negative GAAIS (F2)	AI10	Organisations use Artificial Intelligence unethically	0.325	Removed	0.888
	AI14	I think artificially intelligent systems make many errors	0.398	Removed	
	AI15	I find Artificial Intelligence sinister	0.827	0.815	
	AI16	Artificial Intelligence might take control of people	0.758	0.761	
	AI17	I think Artificial Intelligence is dangerous	0.870	0.876	
	AI18	I shiver with discomfort when I think about future uses of Artificial Intelligence	0.793	0.795	
	AI19	People like me will suffer if Artificial Intelligence is used more and more	0.661	0.668	
	AI20	Artificial Intelligence is used to spy on people	0.606	0.599	

CR = composite reliability; GAAIS = General Attitudes Towards Artificial Intelligence Scale

## Data Availability

The data that support the findings of this study are available from the corresponding author upon reasonable request.
